# CHARTING THE POST-COVID-19 COURSE IN NURSING HOMES THROUGH STAKEHOLDER ENGAGEMENT AND ADVOCACY

**DOI:** 10.1093/geroni/igac059.056

**Published:** 2022-12-20

**Authors:** Thomas Caprio, Brian Lindberg

## Abstract

Nursing homes sustained a disproportionate number of negative effects from the COIVD-19 pandemic including strained resources, significant decline in workforce capacity, high prevalence of infections and mortality among older adults, and a need for ongoing support and timely information related to COVID-19 vaccination and boosters. The HRSA-funded Geriatric Workforce Enhancement Programs (GWEPs) have responded to the growing need to support nursing homes in the recovery phase of the COVID-19 pandemic. Several of these GWEPs undertook new efforts under supplemental federal funding to conduct outreach and partner with nursing home stakeholders including direct care workers, residents, families, and community-based organizations to address gaps in education and training, to promote age-friendly care, and to address social determinants of health. In this symposium GWEPS from different geographic areas share their experiences in developing nursing home focused interventions, engaging feedback to refine curricula, and identify areas to advocate for future policy considerations to support and enhance nursing homes post-pandemic.

